# Surgical outcomes of intermittent exotropia according to exotropia type based on distance/near differences

**DOI:** 10.1371/journal.pone.0214478

**Published:** 2019-03-25

**Authors:** Gi Hyun Bae, Seok Hyun Bae, Dong Gyu Choi

**Affiliations:** 1 Department of Ophthalmology, Sahmyook Medical Center, Seoul, Korea; 2 Department of Ophthalmology, Hallym University College of Medicine, Kangnam Sacred Heart Hospital, Seoul, Korea; Faculty of Medicine, Cairo University, EGYPT

## Abstract

We compare the surgical outcomes of intermittent exotropia of the basic, pseudo-divergence excess (pseudo-DE) and true divergence excess (true DE) types. A study was performed with 342 patients who had undergone surgery for intermittent exotropia of the basic, pseudo-DE or true DE type with a postoperative follow-up period of 6 months or more. The main outcome measures were postoperative angles of deviation at distance and near, and surgical success rates. Surgical success was defined as alignment between exodeviation of 10 PD and esodeviation of 5 PD at distance and near. Additionally, survival curves of recurrence were analyzed by the Kaplan-Meier method. The postoperative angles of deviation at both distance and near in pseudo-DE type were significantly smaller than those in basic type at the final examination (p = 0.003, <0.001). The final surgical success rate in pseudo-DE (70.2%) was better than in basic (46.3%) or true DE (28.6%) (p = 0.003, 0.01). Reoperation for recurrent exotropia was performed in 27% of the basic, 17% of the pseudo-DE, and 35.7% of the true DE cases. According to a survival analysis for recurrence, patients with pseudo-DE showed lower incidence of recurrence than did patients with basic and true DE (p = 0.003, 0.02). In conclusion, the patients with intermittent exotropia of the pseudo-DE type showed better surgical outcomes than those with the basic or true DE type. Pseudo-DE also showed a lower recurrence rate than did the other 2 groups.

## Introduction

Intermittent exotropia could be classified considering to the disparity of distance and near deviation. Based on the Burian’s classification, 1) patient in whom the distance deviation equals or less than 10PD of near is defined as basic type, 2) the distance deviation is more than 10PD larger than near deviation as true divergence excess (true DE) type, 3) the near deviation increases within 10PD of distance after monocular occlusion as simulated divergence excess or pseudo-divergence excess (pseudo-DE) type. 4) the near deviation is larger than distance as convergence-insufficiency [1–4].

In measurements of the deviation, monocular occlusion should be made to eliminate fusional impulses. Scobee[5] proposed 24 hours of monocular occlusion and Burian[1] suggested that only 30 to 45 minutes of monocular occlusion was enough to remove fusional stimuli. He classified such patients as having pseudo-DE, in which patients had equal deviations at near and distance after monocular occlusion test. Recently, Kushner[6, 7] used the term ‘‘tenacious proximal fusion” for such a persistent convergence innervation at near fixation.

Previously different surgical procedures were recommended based on the type of exotropia[1–4, 6]. Burian et al.[1–4] recommended unilateral recess/resect surgery for the basic and simulated DE type and bilateral lateral rectus recess surgery for the true DE type. Hardesty et al.[8] suggested that the similar results of bilateral recession of lateral rectus muscles in patient with the divergence excess and basic type of exotropia. Later, Kushner[6] showed that patients with the basic type had better outcomes with recess/resect procedure than symmetrical lateral rectus recessions, and patients with the pseudo-DE did well with symmetrical lateral rectus recessions. Despite of several studies[1–4, 6, 8], there has not been a definite standard for selection of surgical method according to the type of exotropia.

The mechanisms of exotropia should be considered in selection of surgical methods, which could affect the outcomes.[7–9] So many investigators had tried to determine mechanisms for developing each type of exotropia.[7, 10, 11] Kushner[7] explained the “distance/near differences” in divergence excess type by the “tenacious proximal fusion(TPF)”, accommodative convergence to accommodation(AC/A) ratio, and proximal convergence. And he suggested that exodeviation in pseudo-DE and true-DE types would manifest via different mechanism.

In this study, we supposed that the pseudo-DE and true DE types would have different surgical outcomes if each type is attributed to different mechanisms. The purpose of the present study was to compare the surgical outcomes of pseudo-DE and true DE type, and furthermore, to compare them with basic type of intermittent exotropia.

## Materials and methods

### 1. Study design and participants

We retrospectively reviewed the medical records of 355 patients who had undergone surgery for intermittent exotropia with a minimum postoperative follow-up of 6 months. We excluded 13 patients with the convergence-insufficient type of exotropia in order to focus on the surgical outcomes for the basic, pseudo-DE and true DE types of intermittent exotropia; the final cohort, accordingly, numbered 342 patients. Patients with sensory exotropia resulting from unilateral visual impairment, history of prior strabismus surgery, limitation of extraocular movement and neurologic disorders, as well as those for whom the follow-up period was less than 6 months postoperatively, had been excluded from the initial medical records review. The study was approved by the Institutional Review Board of the Hallym University Medical Center. The IRB waived the requirement for informed consent.

### 2. Preoperative examination

Refractive errors were determined by cycloplegic refraction with topical administration of 1% cyclopentolate hydrochloride (Cyclogyl, Alcon Lab. Inc., Fort Worth, TX, USA) and 1% tropicamide (Mydriacyl, Alcon Lab. Inc.). As described in detail previously[12], one ophthalmologist (DGC) assessed the angle of deviation by alternate prism cover test with accommodative targets for fixation at distance (6 m) and near (33 cm) with best optical correction. We used a modified Krimsky method for a few uncooperative patients. If the exodeviation at distance was larger than 10 PD compared with that at near, one eye was occluded for 1 hour in order to eliminate fusional convergence, after which the alternate prism cover test was repeated at near and distance. Additional postocclusion near measurement with +3.0 D sphere was performed before allowing the patients to restore binocular fusion. We grouped the patients according to the distance/near deviation difference (Burian’s classification)[1]: 1) the basic type, if the distance deviation was within 10 PD of the near deviation; 2) the pseudo-DE type, if the distance exodeviation was larger than the near, but the near deviation increased within 10 PD of the distance deviation after 1-hour monocular occlusion; 3) the true DE type, if the deviation was at least 10 PD larger at distance than at near, even after 1-hour occlusion; (4) the convergence-insufficiency type, if the near deviation was at least 10PD greater than the distance deviation. The following patient preoperative characteristics were analyzed: gender, age at onset, age at diagnosis, age at surgery, duration from onset to diagnosis, duration from onset to surgery, refractive errors, amblyopia, deviation at distance and near, associated strabismus (vertical deviation, dissociated vertical deviation [DVD], A or V pattern, and oblique muscle dysfunction), lateral incomitance, fixation preference, stereopsis, and surgical methods employed. Fixation preference was determined by repeated cover-uncover testing. Lateral gaze incomitance was defined as ≥10 PD change in the right or left gaze relative to the primary position. Refractive errors were determined by cycloplegic refraction with topical administration of 1% cyclopentolate hydrochloride (Cyclogyl, Alcon Lab. Inc., Fort Worth, TX, USA) and 1% tropicamide (Mydriacyl, Alcon Lab. Inc.). Amblyopia was defined as more than a two-line difference between the eyes or lower than 20/30 in best-corrected visual acuity. Sensory status was evaluated by the Titmus Stereotest (Stereo Optical Co., Inc., Chicago, IL, USA) and, with cooperative patients, the Worth 4-Dot test as well. Stereoacuity of ≤100 seconds of arc was defined as good stereopsis.

### 3. Strabismus surgery

All surgical procedures were performed under general anesthesia by the same surgeon (DGC), and the surgical dose was determined based on the angle of distant exodeviation using Park’s modified formula (Table 1)[13] as described in detail previously [12]. Selection of the method of surgery was made by the surgeon, who had no preference between bilateral lateral rectus recession (BLR) and unilateral lateral rectus recession and medial rectus resection (RR) on the non-dominant eye. Most patients with exotropia of less than 25 PD at distant and near fixation underwent unilateral lateral rectus recession (ULR).

**Table 1:** Surgical dosages for intermittent exotropia patients.

Distance deviation (PD)	BLR (mm)	RR (mm)	ULR (mm)
15	4.0	4.0/3.0	8.0
20	5.0	5.0/4.0	9.0
25	6.0	6.0/5.0	10.0
30	7.0	7.0/5.5	
35	7.5	7.5/6.0	
40	8.0	8.0/6.5	
50	9.0	9.0/7.0	

PD = prism diopters, BLR = bilateral lateral muscle recession, RR = unilateral lateral recuts muscle recession and medial rectus muscle resection, ULR = unilateral lateral rectus recession

### 4. Postoperative measurement

Postoperative alignment at distance and near was recorded at 1, 3, 6, and 12 months postoperatively and at final follow-up. Patients with diplopia with postoperative esotropia were managed by full-time alternate patching for 1–4 weeks until the diplopia or esodevation was resolved. If the esotropia persisted for several months postoperatively, residual hyperopia was corrected after cycloplegic refraction and base-out Fresnel press-on prisms (3M Press-On Optics 3M Health Care, St Paul, MN, USA) were prescribed.

### 5. Main outcomes

The main outcome measures were postoperative angles of deviation at distance and near and surgical success rates at 1, 3, 6, and 12 months postoperatively and at final follow-up according to the basic, pseudo-DE, and true DE types. In reoperation cases, the last visit before reoperation was considered as the final follow-up for the assessment of surgical outcome. Surgical success was defined as an alignment between exodeviation of 10 PD and esodeviation of 5 PD at distance and near. Overcorrection was defined as esodeviation of >5 PD, and undercorrection or recurrence as exodeviation of >10 PD. Also, the three groups’ survival curves of postoperative recurrence were compared.

### 6. Statistical analysis

Statistical analyses were performed using PASW 20.0 (SPSS Inc, Chicago, IL USA). The Pearson chi-square test and Fisher’s exact test were used for qualitative data (including gender, amblyopia, associated strabismus, lateral incomitance, fixation preference, stereopsis, surgical method, success rate). The Kruskal-Wallis test and Mann-Whitney U test with Bonferroni’s correction were used for quantitative data (including age at onset, age at diagnosis, age at surgery, duration from onset to diagnosis, duration from onset to surgery, refractive errors, and pre- & postoperative angles of deviation) Kaplan-Meier survival analysis was used to estimate survival curves of recurrence. Differences in survival curves among the groups were analyzed with the log-rank test. A *p*<0.05 was considered to be statistically significant.

## Results

### 1. Preoperative characteristics of patients

Of the 355 patients who had undergone surgery for intermittent exotropia, 13 with the convergence insufficiency type of exotropia were excluded. Thus, a total of 342 patients divided into 3 groups, the basic type (281 patients), the pseudo-DE type (47 patients), and the true DE type (14 patients), were included in the analysis.

The preoperative patient characteristics in the 3 groups are summarized in Table 2. The ratio of males to females was 121:160 in basic, 22:25 in pseudo-DE, and 5:9 in true DE (*P* = 0.7). The mean age at onset was 4.0±3.1 years in basic, 3.8±2.9 in pseudo-DE, and 3.0±2.4 in true DE (*P* = 0.5); the mean age at surgery was 6.2±3.1, 6.2±2.6, and 6.0±1.7 years, respectively (*P* = 0.9). The preoperative mean angles of exodeviation at distance were 27.2±8.1 PD in basic, 27.4±5.6 in pseudo-DE, and 28.0±4.2 in true DE, which differences were not significant (P = 0.3). The mean angles of exodeviation at near were 27.9±8.6 PD, 26.9±6.9, and 15.4±5.6, respectively, which showed significantly smaller angle of exodeviation at near in true DE compared with the other two groups (P<0.001). As for the surgical methods, RR was the main surgical method in basic (70.1%) and pseudo-DE (70.2%); however, BLR was performed in 64.3% of true DE cases (P<0.001). The postoperative follow-up periods of the 3 groups were 50.6±40.9, 43.7±34.0, and 58.5±65.8 months, respectively (p = 0.4).

**Table 2:** Preoperative characteristics of patients.

	Basic (n = 281)	Pseudo-DE (n = 47)	True DE (n = 14)	*p* value
Male: Female	121:160	22:25	5:9	0.7*
Age at onset (years)	4.0±3.1 (0.5~18.6)	3.8±2.9 (0.7~11.4)	3.0±2.4 (0.8~9.3)	0.5†
Age at diagnosis (years)	5.5±3.3 (0.5~19.0)	5.1±2.7 (0.9~11.4)	5.2±2.3 (1.1~9.4)	0.9†
Age at surgery (years)	6.2±3.1 (0.8~19.1)	6.2±2.6 (1.4~13.1)	6.0±1.7 (3.1~ 9.8)	0.9†
Duration from onset to diagnosis (months)	18.7±25.9 (0~204)	17.7±18.1 (0~75)	26.9±22.0 (1~82)	0.1†
Duration from onset to surgery (months)	26.8±26.2 (6~205)	29.7±23.8 (6~104)	36.0±21.8 (5~83)	0.1†
Refractive errors				
Dominant eye (SE, diopters)	0.04±1.6 (-5.5 ~ +7.0)	-0.3±2.0 (-11.0 ~ +1.5)	0.6±2.1 (-2.3 ~ +6.5)	0.4†
Non-dominant eye (SE, diopters)	0.03±1.8 (-7.0 ~ +7.0)	-0.03±1.2 (-2.3 ~ +3.8)	0.4±2.6 (-5.8 ~+6.0)	0.4†
Amblyopia	30/251(12.0%)	5/42(11.9%)	2/13(15.4%)	0.7‡
Preoperative angle of exodeviation (PD)					
Distance	27.2±8.1 (15~70)	27.4±5.6 (18~40)	28.0±4.2 (20~35)	0.3†
Near	27.9±8.6 (10~65)	26.9±6.9 (15~45)	15.4±5.6 (9~25)	<0.001†
Associated features				
Vertical deviation	60 (22.3%)	11 (23.4%)	8 (57.1%)	0.02‡
DVD	22 (7.8%)	0 (0%)	2 (14.3%)	0.2‡
AV pattern	18 (6.7%)	2 (4.3%)	2 (14.3%)	0.3‡
Oblique muscle dysfunction	69 (24.8%)	8 (17.0%)	4 (28.6%)	0.4‡
Lateral incomitance	7 (2.5%)	2 (4.3%)	1 (7.1%)	0.5‡
Fixation preference	164 (60.3%)	36 (78.3%)	9 (64.3%)	0.1‡
Good stereopsis (≤100 seconds)	148/189 (78.3%)	25/35 (71.4%)	5 /10 (50.0%)	0.1‡
Fusion on worth-4-dots	67/189 (35.3%)	15/32 (46.9%)	5/10 (50%)	0.3‡
Surgical method				
R&R	197 (70.1%)	33 (70.2%)	3 (21.4%)	<0.001‡
BLR	29 (10.3%)	9 (19.1%)	9 (64.3%)	
ULR	55 (16.1%)	5 (10.6%)	2 (14.3%)	
Postoperative follow-up period (months)	50.6±40.9 (6–219)	43.7±34.0 (6~131)	58.5±65.8 (6~190)	0.4†

DE = divergence excess type, SE = spherical equivalent, PD = prism diopters, DVD = dissociated vertical deviation, RR = unilateral lateral recuts muscle recession and medial rectus muscle resection, BLR = bilateral rectus muscle recession, ULR = unilateral lateral rectus muscle resection

* Pearson chi square test

† Krunskal-Wallis test

‡ Fisher’s exact test

### 2. Postoperative angles of deviation

The postoperative angles of deviation at final follow-up were 11.8 ± 10.3, 6.0± 12.0, and 11.3 ± 17.5 PD at distance and 12.4 ± 11.7, 4.9 ± 11.8, and 6.3 ± 15.6 PD at near in basic, pseudo-DE, and true DE, respectively (Tables 3 and 4). The postoperative deviations at both distance and near in pseudo-DE were significantly smaller than in the basic type (p = 0.003, <0.001). However, there were no differences between basic and true DE or between pseudo-DE and true DE.

**Table 3:** Postoperative angles of deviation (PD) at distance.

	mean±SD			P value*		
	Basic (n = 281)	Pseudo-DE (n = 47)	True DE (n = 14)	Basic vs. Pseudo-DE	Basic vs. True DE	Pseudo-DE vs. True DE
1 day	-4.2 ± 6.3	-3.9 ± 5.3	-2.7 ± 5.1	1.0	1.0	1.0
1 week	-0.8 ± 5.1	-1.6 ± 5.7	-1.3 ± 2.6	0.9	1.0	1.0
1 month	1.4 ± 4.9	0.7 ± 3.5	1.7 ± 2.9	0.8	1.0	0.3
3 months	2.9 ± 5.6	1.1 ± 4.7	2.9 ± 6.9	0.05	1.0	1.0
6 months	4.3 ± 6.5	2.1 ± 6.6	5.6 ± 9.0	0.10	1.0	0.7
1 year	5.0± 7.7	6.1 ± 7.5	5.7 ± 7.9	1.0	1.0	1.0
Final F/U	11.8 ± 10.3	6.0± 12.0	11.3 ± 17.5	**0.003**	1.0	0.2

PD = prism diopters, DE = divergence excess type

A minus angle of deviation indicates esotropia, and a plus angle exotropia.

* Mann-Whitney U test with Bonferroni’s correction

**Table 4:** Postoperative angles of deviation (PD) at near.

	mean±SD			P value*		
	Basic (n = 281)	Pseudo-DE (n = 47)	True DE (n = 14)	Basic vs. Pseudo-DE	Basic vs. True DE	Pseudo-DE vs. True DE
1 day	-3.0 ± 6.3	-4.3 ± 6.3	-3.2 ± 3.9	0.5	0.9	1.0
1 week	-1.2±4.7	-2.5 ± 6.0	-2.2± 3.9	0.1	0.4	1.0
1 month	1.0± 4.7	-0.1 ± 4.5	-0.5 ± 3.8	0.4	0.3	1.0
3 months	2.7 ± 5.7	0.8 ± 5.0	-0.6 ± 5.3	**0.02**	0.3	1.0
6 months	4.3 ± 6.4	0.6 ± 4.8	0.3 ± 11.4	**0.006**	1.0	1.0
1 year	5.0±8.2	3.4 ± 6.6	1.4 ± 3.8	0.5	0.6	1.0
Final F/U	12.4 ± 11.7	4.9 ± 11.8	6.3 ± 15.6	**<0.001**	0.6	0.9

PD = prism diopters, DE = divergence excess type

A minus angle of deviation indicates esotropia, and a plus angle exotropia.

* Mann-Whitney U test with Bonferroni’s correction

### 3. Surgical success rates

Until postoperative 1 year, the surgical success rates did not differ by exotropia type. At the final examination though, the surgical success rates were 46.3, 70.2, and 28.6% in basic, pseudo-DE, and true DE, respectively, which results served to highlight the better outcome for the pseudo-DE type relative to the others (P = 0.003, 0.01, Table 5).

**Table 5:** Surgical success rates by intermittent exotropia types.

		mean±SD		P value*		
	Basic (n = 281)	Pseudo-DE (n = 47)	True DE (n = 14)	Basic vs. Pseudo-DE	Basic vs. True DE	Pseudo-DE vs. True DE
1 month	88.6%	93.6%	100%	0.4	0.4	1.0
3 months	83.6%	89.4%	71.4%	0.4	0.3	0.2
6 months	77.2%	80.9%	64.3%	0.7	0.3	0.3
1 year	67.8%	66.7%	66.7%	1.0	1.0	1.0
Final F/U	46.3%	70.2%	28.6%	**0.003**	0.3	**0.01**

PD = prism diopters, DE = divergence excess type

Surgical success: ≤10 PD of exotropia ~ ≤5 PD of esotropia

* Pearson chi square test

### 4. Reoperation rates

Among the total of 342 patients, 96 (28.07%) underwent an additional procedure 51.33±33.30 months after the first operation. Operations for recurrent exotropia were performed in 76 basic patients (27%), 8 pseudo-DE (17%) patients, and 5 true DE patients (35.7%). One basic patient, 1 pseudo-DE patient and 1 true DE patient underwent reoperation for consecutive esotropia. One basic patient underwent surgery for DVD, and 1 basic and 2 true DE patients were operated on for superior oblique palsy.

### 5. Survival analysis for recurrence

**Fig 1:**
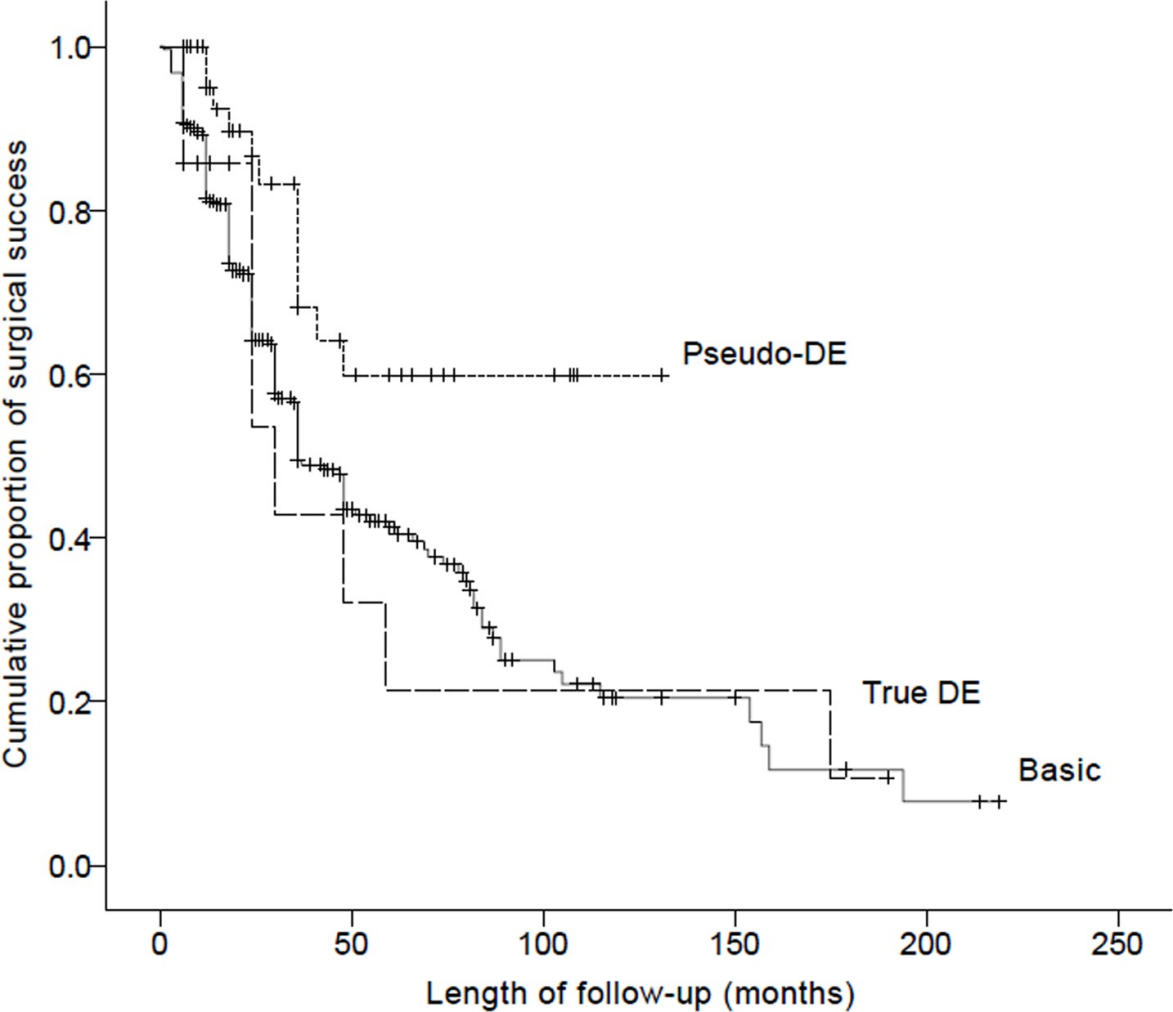
Survival curves for recurrence in basic, pseudo-DE, and true DE types of intermittent exotropia. Pseudo-DE showed significantly lower recurrence compared with basic and true DE (P<0.003, 0.02, log rank test).

Until the final follow-up, 174 patients (50.9%) showed recurrence; the mean time from the first surgery to recurrence was 32.6±32.9 months. According to the types of intermittent exotropia, 153 basic (54.4%), 12 pseudo-DE (25.5%), and 9 true DE patients (64.3%) suffered recurrence after the first surgery. In a comparison of the survival curves for recurrence, the pseudo-DE type showed a significantly lower rate compared with the basic and true DE types (P<0.003, 0.02 by log-rank test, Fig 1).

## Discussion

There have been many reports on evaluations of surgical outcomes and clinical courses in intermittent exotropia patients.[6, 12, 14–18] However, only a few studies have compared results according to exotropia types including pseudo-DE and true DE. Our present study compared the surgical outcomes of the basic, pseudo-DE, and true DE types of intermittent exotropia. First, pseudo-DE showed better final surgical success at the final examination than did the 2 other groups. Second, the postoperative exodeviations at both distance and near in pseudo-DE were significantly smaller compared with the basic type. Third, pseudo-DE showed lower incidence of recurrence from the first surgery compared with the 2 other groups.

Keenan and Willshaw[15] reviewed 42 patients, including 11 (26.2%) with constant exotropia, 17 (40.5%) with simulated distance exotropia, and 14 (33.3%) with true distance exotropia. They reported that 63.6% with constant exotropia, 70.6% with simulated distance exotropia, and 85.7% with true distance exotropia showed less than ±15 PD 3–6 months postoperatively. Pineles et al.[18] reviewed the long-term (>10 year) surgical outcomes for 50 patients with intermittent exotropia. They reported that 64% of patients had excellent motor alignment, but only 38% according to combined motor/sensory criteria. In their subgroup analysis, 41% (14 of 34) of the basic type, 57% (4 of 7) of the pseudo-divergence excess type, and 11% (1 of 9) of the convergence-insufficiency type manifested excellent motor/sensory outcomes. These results are comparable with our study, which showed a higher final success rate in the pseudo-DE group than in the other 2 groups. Thirty of Pineles et al.’s patients of the total of 50 (60%) required additional strabismus surgical procedures a mean 7.4 years after the initial surgery. For the convergence insufficiency, basic, and pseudo-divergence excess patients, 67, 52, and 43%, respectively, underwent reoperation. These results are very meaningful to us, because they reflect an analysis of surgical outcomes and long-term clinical courses according to exotropia types. Nonetheless, the previous investigations, when compared with the present study, have some limitations. The outcomes of surgery for intermittent exotropia, for example, were quite variable, due to differences among the criteria used to define surgical success, differences among surgical methods, and variations in follow-up periods. Keenan and Willshaw[15] included not only cases of intermittent exotropia but also of other forms of constant exotropia including infantile exotropia. Moreover, they defined favorable outcome as a final alignment, for near and distance, within 10 PD of straight, or within 20 PD of straight with evidence of binocular single vision. Pineles et al.[18] included patients who were followed-up on for more than 10 years, which was much longer than in our study (62.78±49.90 months after the first strabismus operation). Also, their cohort did not include any patients with the true divergence excess type of exotropia.

Kushner[7] reported surgical outcomes for basic and simulated divergence excess intermittent exotropia patients. Among the basic exotropia cases, the patients who had undergone RR showed better outcomes than those who had undergone BLR (82 vs. 52%). Meanwhile, 80% of simulated divergence excess cases showed a favorable outcome after BLR. This study did not directly compare surgical success between the 2 groups. However, its data suggested that patients with pseudo-DE had better or at least similar surgical success to those basic type, which is comparable to ours.

In our present study, the final exodeviation angles at both distance and near in pseudo-DE were significantly smaller than those in the basic type. According to the survival analysis for recurrence, pseudo-DE showed lower incidence of recurrence relative to the 2 other groups. These results suggest that patients with pseudo-DE have less exotropic drift after surgery.

Our study has some limitations, First, this was a retrospective study, so we performed much more RR than BLR in basic and pseudo-DE patients, and BLR than RR in true DE patients although we had no intended preference in selecting surgical methods. Therefore, further prospective randomized controlled studies with standardization of the surgical technique are needed.

Additionally, measuring the angle at only 6 meters is not a true distant angle and distance deviation may be increased with distant outdoor target fixation.[19] Patients who underwent surgery for the angle at 6 meters could have more undercorrection, which also have impact on the surgical success rate. Unfortunately, it’s not easy to measure the angle at more than 6 meters in our clinical setting. However, core results and conclusion of this paper (pseudo-DE type showed better final success rate and lower recurrence than those with the basic or true DE) might be consistent.

## Conclusions

Patients with the pseudo-DE type of intermittent exotropia showed better surgical outcomes and lower incidence of recurrence compared with the basic and true DE types. Further prospective study with a larger number of patients and standardized criteria for surgical methods are needed.

## Supporting information

S1 Dataset Law dataset of the subjects. (XLS)
